# Phenotypic Plasticity in Reproductive Traits of the Perennial Shrub *Ulex europaeus* in Response to Shading: A Multi-Year Monitoring of Cultivated Clones

**DOI:** 10.1371/journal.pone.0137500

**Published:** 2015-09-18

**Authors:** Anne Atlan, Benjamin Hornoy, Florian Delerue, Maya Gonzalez, Jean-Sébastien Pierre, Michèle Tarayre

**Affiliations:** 1 UMR 6553 ECOBIO, CNRS/University of Rennes 1, Rennes, France; 2 CEF, University of Laval, Montréal, Quebec, Canada; 3 Bordeaux INP, G&E, EA 4592, Pessac, France; Beijing Forestry University, CHINA

## Abstract

Phenotypic plasticity may be advantageous for plants to be able to rapidly cope with new and changing environments associated with climate change or during biological invasions. This is especially true for perennial plants, as they may need a longer period to respond genetically to selective pressures than annuals, and also because they are more likely to experience environmental changes during their lifespan. However, few studies have explored the plasticity of the reproductive life history traits of woody perennial species. This study focuses on a woody shrub, *Ulex europaeus* (common gorse), and on the response of its reproductive traits to one important environmental factor, shading. The study was performed on clones originating from western France (within the native range of this invasive species) and grown for seven years. We compared traits of plants grown in a shade treatment (with two successive shade levels) vs. full natural light. The traits monitored included flowering onset, pod production and seed predation. All traits studied responded to shading, exhibiting various levels of plasticity. In particular, dense shade induced a radical but reversible decrease in flower and pod production, while moderate shade had little effect on reproductive traits. The magnitude of the response to dense shade depended on the genotype, showing a genetically based polymorphism of plasticity. The level of plasticity also showed substantial variations between years, and the effect of environmental variations was cumulative over time. This suggests that plasticity can influence the lifetime fitness of *U*. *Europaeus* and is involved in the capacity of the species to grow under contrasting environmental conditions.

## Introduction

Phenotypic plasticity, i.e. the capacity of a single genotype to change its phenotype in response to the environment, determines the range of conditions under which an individual can survive and reproduce. Together with genetic evolution, phenotypic plasticity allows individuals and populations to cope with environmental variations in both time and space, and is thus critical in the context of global change [1,2,3]. In addition, adaptation and/or plasticity can be involved in the colonizing success of many plant invaders, allowing them to thrive in a wide range of exotic environments [4,5]. Genetic evolution can be very rapid, as shown by studies of geographical clines for invasive species present in a wide range of climates [6,7,8]. However, the selection of genes adapted to the new environmental conditions generally requires more than one generation, and cannot be the only factor favouring the adaptation of the first migrants. This is why plasticity is considered as an important determinant of pioneer species establishment and species invasiveness [9,10].

Plasticity itself can have a genetic basis and can be selected for at a local scale, either by fine-grained spatial heterogeneity [11,12] or by temporal fluctuations of the environment within one generation [13, 14]. Plasticity is expected to be of primary importance for species experiencing environmental changes within one generation, and especially for long-lived woody perennials [13,14,15,16]. Plasticity is also beneficial for pioneer species, which establish in open environments, by permitting survival and reproduction later in the succession, when the canopy closes and light availability diminishes [17,18]. Variation in light availability has been shown to have direct impacts on several life history traits, such as growth pattern, flower production and flowering phenology [19,20,21]. Light availability can also have indirect effects on plant reproductive success through plant-insect interactions. Firstly, light conditions have been shown to affect the foraging behaviour of pollinators [22] and phytophagous insects [23]. Secondly, light conditions could induce changes in plant traits that affect insect behaviour, such as floral display [24]. Plasticity in such traits could thus allow the maintenance of long-lived pioneer species and their reproduction later in the succession by maximizing their lifetime fitness.

Although plasticity to such environmental variation is important in woody perennials, especially in the context of biological invasion or climate change [25], most studies of plasticity have dealt with herbaceous plants and/or annuals (see for example the review by Matesanz [26]. In addition, among studies that investigated plasticity in woody species, the main focus was on the vegetative traits of seedlings [27,28,29] and very few studies examined reproductive traits (but see [16]). Indeed, exploring plasticity of reproductive traits in perennial woody species is difficult because it requires the growth of sexually mature plants, which can take several years, and necessitates long-term monitoring of potentially large individuals in controlled environments.

Here we present a multi-year monitoring of plasticity in a perennial woody shrub, *Ulex europaeus* (common gorse), in response to shading. Individuals of this species live up to 20 years and can reach four metres high [30]. As they reproduce from their third year, long-term monitoring of their adult stage is possible. *Ulex europaeus* is native to Western Europe, where it can be found in open habitats such as heathlands and fallow land, or in more closed habitats later in the succession, such as young forests [31,32]. *Ulex europaeus* has been introduced throughout the world and has become invasive in a wide range of latitudes and altitudes [33]. *Ulex europaeus* can face environmental conditions with contrasting light availability both in its native and invasive ranges, and phenotypic plasticity in response to shading may thus be an important trait of the species. We explored this plasticity on plants from Brittany (Western France), a region of its native range where the phenotypic and genetic variability of life history traits was studied previously [34,35].

This study was made in a common garden where light availability varied in space (a shade-house covered half the garden) and time (the type of shade-house and the weather varied between years). We developed a protocol to clone *U*. *europaeus* individuals, and then compared plants of different genotypes to investigate the genetic basis of the responses to variations in light availability. We focused on the plastic response of flowering phenology, pod density and sensitivity to seed predation, three traits that have been shown to be highly variable between and within native populations of *U*. *europaeus*. The genetic basis of this variability was demonstrated by experimental studies [35], but plastic responses to environmental variation were also suggested during the long-term monitoring of natural populations [34].

The present study, in a controlled environment, complements another recent study on the plasticity of growth and fecundity of *U*. *europaeus* plants in natural populations [32]. We focused on traits likely to influence the adaptation of *U*. *europaeus* to variable levels of light exposure. More precisely, we aimed to answer the following questions: 1/ How does shading affect traits related to fecundity and seed predation in *U*. *europaeus*? 2/ How do temporal variations interact with the response to shading? 3/ Does the observed plastic response depend on plant genotype?

## Materials and Methods

### The biological model

*Ulex europaeus* (Fabaceae, Genisteae) is an evergreen hermaphrodite shrub, widespread along the Atlantic coasts of Europe. Since the 18^th^ century it has been introduced in several countries including USA, Chile, South Africa, Hawaii, Reunion, Australia and New-Zealand, where it is often considered as a noxious invasive species [33]. It is a pioneer and light-demanding species [30] that commonly forms dense thickets in areas with full light exposure. In its zone of origin, its preferred habitats are heathlands and fallow land, but it can also be found in the undergrowth [31] and in forest hedges [32]. In the invasive range, it is found preferentially in open environments, but also in forest hedges or clearings.

Plants begin to flower at the age of three and the flowering season lasts from September to May, with a peak in April. Two main flowering types coexist in all natural populations: long-flowering plants that flower from autumn to spring and partly escape seed predation over time, and short-flowering plants, that flower only in spring and reduce seed predation by mass flowering [34,35]. Flowers remain open for up to three weeks, ensuring successful pollination by a diversity of bees including bumblebees, even during winter [36]. Plants differ in their time of flowering onset, but fructification is more synchronous and pod ripening occurs in May-June for most individuals [34,35].

*Ulex europaeus* pods can be infested by three types of insect [37]: the weevil *Exapion ulicis* (Curculionidae) is a seed predator specific to *U*. *europaeus*. Females lay eggs inside the pods, larvae feed on the seeds, and adult weevils are released at pod dehiscence. The weevil can be attacked by a parasitoid wasp, the hymenopteran *Pteromalus sequester* (Pteromalidae), which develops in the larvae of *E*. *ulicis*. Larvae of the moth *Cydia succedana* (Tortricidae) also develop within pods, but they are able to bore holes to leave the pods by themselves. Its past presence is typically indicated by a hole and excrements.

### Experimental design

We grew 50 ramets (five maternal genotypes and 10 ramets per genotype). The mother plants came from an experimental garden located in the campus of Rennes University. Plants of this garden were grown from seeds collected in natural populations of Brittany in 2001, germinated in the same year and planted in 2002 (see [35] for details). In order to increase the diversity of the maternal genotypes, we chose mother plants from two different populations (referred to as "Château Vaux" and "Pointe Meinga" in [35]). Of the five mother plants chosen, three had a long-flowering phenotype and were called L1, L2 and L3, and two had a short-flowering phenotype and were called S1 and S2.

Cloning was carried out in September 2005 by taking cuttings from the ends of green shoots. The cut ends were moistened with 0.25% β-indole butyric acid (rooting hormone) before being planted in pots filled with equal proportions of sand, soil and vermiculite. The pots were then placed in a growth chamber and subjected to a photoperiod of 18:6 (day: night) until rooting occurred. Ten ramets per genotype were transplanted into 10 cm diameter pots and grown in a glasshouse for one year. The total number of ramets was limited to 50 due to the size and the surface area covered by an adult plant. These 50 ramets were divided into two groups of 25, each consisting of five randomly chosen ramets of the five genotypes.

In 2006, the ramets were transplanted in a common garden of 200m^2^, located in the campus of Rennes University. The garden was divided in half, and one group of 25 ramets was planted randomly in each half. In July 2007, before the first reproductive season, a shade-house (covered by a black net manufactured to exclude 65% of incident radiation) was installed above half of the garden, to shade one of the two groups. The shade-house was built in the northern half of the garden, and did not shade the group left in full light. Each group thus experienced contrasting environmental conditions, full light *vs* shading. The spatial effect has been tested in a previous experiments performed on gorse grown for 5 years in the same experimental garden [35], and no effect of the position within the garden was detected for any of the variables.

The 65% shading level was chosen because it was approximately the maximum shading under which gorse could be found in the wild [32]. In our study, all plants under 65% of shading survived, but they ceased to produce flowers after two years. In August, 2009, in order to be able to continue the monitoring of reproductive traits under shade, we replaced the shade-house net providing 65% shading by one providing only 30% shading, a level which is tolerated better by gorse [32].

### Measurements

We measured date of flowering onset, pod production, number of seeds per pod and infestation rates by seed predators during four reproductive seasons, from 2007 to 2012. Other measurements were made only once or twice: flower production was measured in spring 2009 and 2010, flower length was measured in spring 2009 and plant height and width were measured in September 2008 (at the highest and larger point of each plant). Due to the death of two ramets, only 48 plants could be fully monitored. All measurements were made following the protocol described in [35].

#### Flowering phenology

The flowering stage of the plants was recorded every two weeks from September to May. We defined 12 flowering and fruiting stages (S1 Text); the first three stages correspond to the swelling of the buds. Flowers may occasionally be present for stage 3, but the flowering onset was attributed to stage 4, when the presence of open flowers is associated to the presence of a majority of large ready-to-open buds. As in all previous studies made on *U*. *europaeus* in Brittany, flowering onset occurred between September to April, but the end of flowering was more synchronous and occurred in April/May. Flowering onset and flowering duration were highly correlated (N = 4 years * 48 plants = 192, R = 0.91, P<0.001), so only the results of flowering onset are presented in this paper.

#### Pod and flower production

When the plants were at their flowering peak (in April) or at their fruiting peak (in June), six shoots per plant were chosen at random and the number of open flowers or ripe pods produced on each was counted, as in [34]. The mean per plant of these six measurements was calculated. The number of flowers and pods per shoot will be referred to as flower density and pod density, respectively.

Flower length was measured in June 2009. The total flower length was measured along the longest axes, from the sepal base to the end of the petal, using a digital calliper. Previous studies [36] had shown that this measurement was strongly correlated with other estimates of flower size (flower width and sepal length). The mean flower length of ramets was estimated by measuring five flowers taken at random on five different shoots per ramet.

#### Number of seeds per pod and seed predation

At each visit we opened 30 random ripe pods, when available, to observe their contents. In each pod we counted the number of seeds and recorded the presence of the different seed predators. The proportion of infested pods was estimated by dividing the number of pods infested by at least one seed predator (i.e. containing weevil, parasitoid, moth or moth traces), by the total number of opened pods. The number of seeds per uninfested pod was calculated from ten pods devoid of any seed predator. Flat or rotten seeds were excluded from the counts. The measurements used for comparisons were those made at the end of June, when the fruiting periods of all flowering types overlapped. Measurements made at this time were found to provide good estimates of the overall infestation rate in spring and of the mean number of seeds per pod [34,35].

### Statistical analysis

Data are provided in file S1 Table. The effects of shading, genotype and year on the variables "flowering onset", "pod density", "seeds per pod" and "proportion of infested pods" were analysed using the function geeglm of the contributed package geepack of the R software [38], i.e. by the combination of the generalized linear model and GEE (Generalized Estimating Equations) estimators. The year effect was declared as a repeated factor on each individual to take into account the possible correlation between the successive measurements made on the same individuals. The type of family error and link function depended on the trait tested, and are provided in the legend of Tables 1 and 2. Some variables were overdispersed, but this is taken into account by the GEE procedure, the choice of “Poisson” for distribution is therefore equivalent to “quasi-Poisson” without gees, and the choice of “binomial” equivalent to “quasi-binomial”. The script is provided in S2 Text. The Analysis of variance or of deviance performed on traits measured a single year were tested with the software SAS [39]: proc GLM for "plant height", "plant width" and "flower length", proc GENMOD with Poisson distribution for "flower density", proc CORR for the Spearman’s rank-order correlations between flower density and pod.

**Table 1:** Deviance analysis made on main reproductive variables of *Ulex europaeus* plants grown in a common garden under 65% shade and in full daylight, in 2007/08 and 2008/09.

shade: 65%		flowering onset		pod density		seeds / pod		rate of infested pods	
		Gaussian		Poisson		Poisson		binomial	
	Df	Chi^2^	P	Chi^2^	P	Chi^2^	P	Chi^2^	P
genotype	4	**213.9**	**<0.001**	13.3	<0.001	**163.3**	**<0.001**	12	<0.05
shade	1	2.2	ns	**109.5**	**<0.001**	14.1	<0.001	55	<0.001
year	1	42.1	<0.001	27.9	<0.001	1.0	ns	90	<0.001
gen x shade	4	23.4	<0.001	26.2	<0.001	33.1	<0.001	**5207**	**<0.001**
gen x year	4	57.5	<0.001	44.6	<0.001	21.5	<0.001	10	<0.05
shade x year	1	0.1	ns	23.3	<0.001	5.3	<0.05	4	<0.1
gen x shade x year	4	4.8	ns	12.2	<0.05	2.03	ns	na	na

N = 48. Df = degree of freedom. The P-value of the total model was P<0.001 for all variables (likelihood ratio test). The error family is noted under each variable name. The links are usual ones: identity for Gaussian, log for Poisson, logit for binomial. In bold type, the factor with the greatest influence—ns: not significant and P>0.10. na = not available (not estimable because too many missing values)

**Table 2:** Deviance analysis made on main reproductive variables of *Ulex europaeus* plants grown in a common garden under 30% shade and in full daylight, in 2009/10 and 2011/12.

shade: 30%		flowering onset		pod density		seeds / pod		rate of infested pods	
		Gaussian		Poisson		Poisson		binomial	
	Df	Chi^2^	P	Chi^2^	P	Chi^2^	P	Chi^2^	P
genotype	4	34	<0.001	**51.7**	**<0.001**	**1268**	**<0.001**	**24.7**	**<0.001**
shade	1	6	<0.05	1.2	ns	1	ns	12.4	<0.001
year	1	**412**	**<0.001**	4.5	<0.05	6	<0.05	0.1	ns
gen x shade	4	16	<0.01	12.7	<0.05	3	ns	15.3	<0.01
gen x year	4	45	<0.001	1.5	ns	23	<0.001	15.2	<0.01
shade x year	1	1	ns	41.7	<0.001	2	ns	0.1	ns
gen x shade x year	4	16	0.0027	11.3	<0.05	na	na	na	na

N = 48. Df = degree of freedom. The P-value of the total model was P<0.001 for all variables (likelihood ratio test). The error family is noted under each variable name. The links are usual ones: Identity for Gaussian, log for Poisson, logit for binomial. In bold type, the factor with the greatest influence—ns: not significant and P>0.10. na = not available (not estimable because too many missing values).

To test the influence of weather on flowering onset, we used a method devised by one of us and improved further by INRA (French National Institute of Agronomic Research) under the acronyme CRITICOR [40]. The data (mean daily temperature, hours of sunshine and precipitation) came from the Rennes Méteo-France weather station, and are available on request. As weather influences are often cumulative and delayed, the model systematically explores the sum or the average obtained for the tested weather variable for a large number of periods each year. It then calculates the correlation coefficient between the values obtained for the different periods and flowering onset for each year from 2007 to 2012. Significant correlation coefficients indicate which weather variables are influential and at what time of year. The statistical test is performed by random sub-sampling (bootstrap, 500 permutations). Were considered as significant all peaks of absolute correlation coefficients values appearing in less than 5% percent of the subsamples. Among those, the largest in absolute values are used to determine the most likely influential period (by its beginning and its duration). A bivariate confidence interval on this critical period can also be obtained by simulation. See CRITICOR notice [41] for more details.

## Results

### General effects of shading, genotype and year

**Fig 1:**
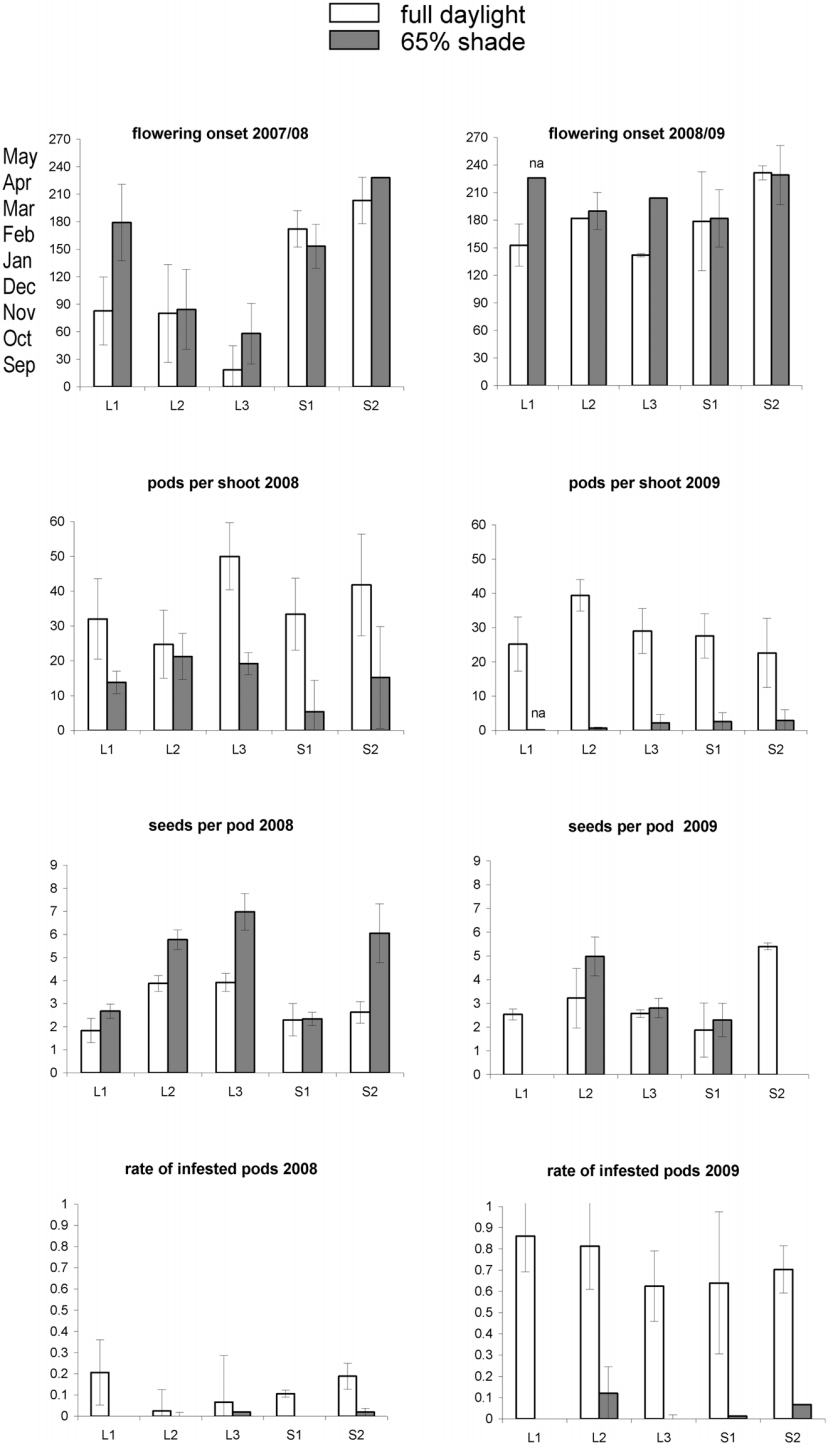
Trait values measured on *Ulex europaeus* in full daylight and under 65% shade. The mean value per genotype (mean of the 5 clones) is given with the standard error. The absence of SE means either that SE = 0, or that SE could not be calculated (na = not available) because only one individual of a given genotype did flower. Dates of flowering onset are given in days, counted from September, 1 (the beginning of the flowering season), and the corresponding months are given for convenience. All other variables were measured in spring.

**Fig 2:**
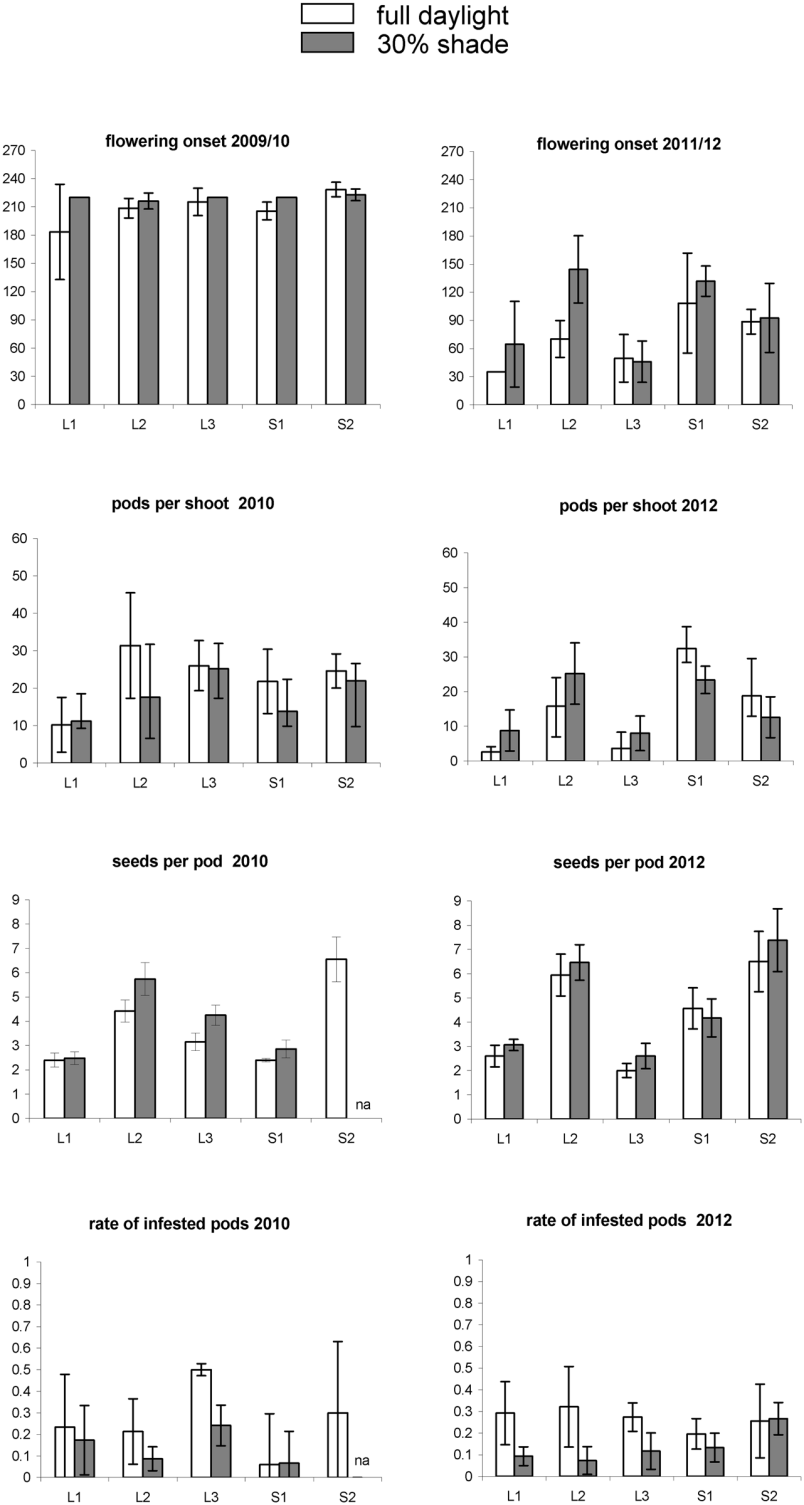
Trait values measured on *Ulex europaeus* in full daylight and under 30% shade. Same legend as Fig 1.

All traits responded to shading (Tables 1 and 2, Figs 1 and 2). For all traits, the magnitude of the response of the main effects and their interactions was higher during the first two years under 65% shading (Table 1), than the following two years under 30% shading (Table 2).

The factor with the greatest influence (the one with the highest F value) depended on the trait. For the date of flowering onset, the most influential factor was the year. For the rate of infested pods, the most influential factor was shading. For pod density and seeds per pod, under 65% shading, shading was the most influential factor, and under 30% shading, genotype was the most influential factor.

### Trait responses to the two shading levels

#### Pod and flower production

Shading tended to decrease pod production and this effect depended mainly on the shading level: with 65% shading (in 2008 and 2009), the number of pods per shoot declined in the first year and fell to almost zero in the second year (Fig 1 and Table 1). With 30% shading (in 2010 and 2012), pod production increased and reached the same level as plants in full daylight (Fig 2 and Table 2).

**Fig 3:**
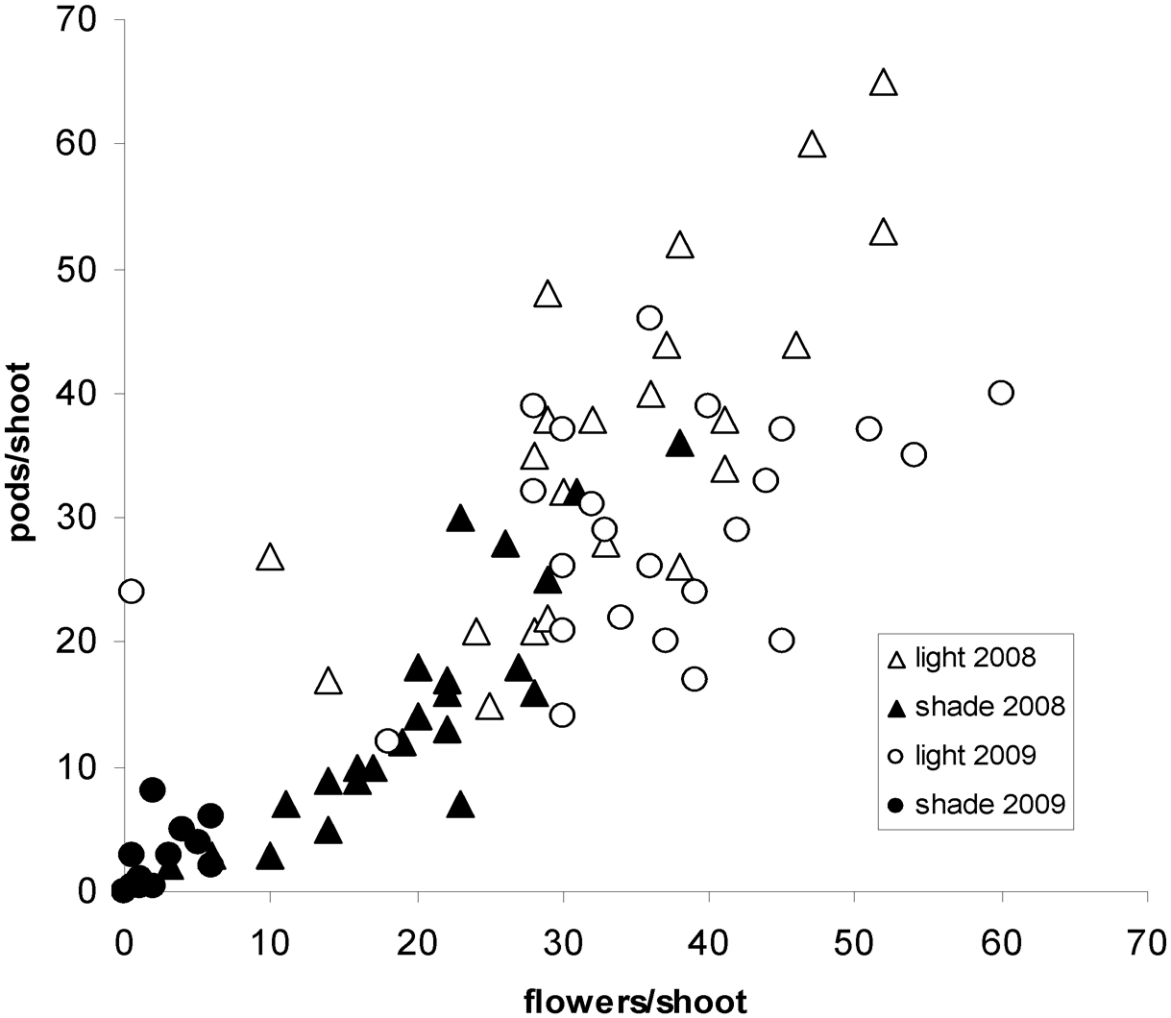
Flower and pod density measured on *Ulex europaeus* in full daylight and under 65% shade.

Flower density was measured in 2008 and 2009 under 65% shade. During this time, shading decreased flower production just as it had decreased pod production (Fig 3): the number of flowers per shoot fell in the first year and became almost zero in the second year. The number of flowers per shoot was strongly correlated with the number of pods per shoot (N = 96, R_Spearman_ = 0.88, P<0.001). In addition, analysis of covariance showed that the ratio pods/flowers was not very dependent of the shading conditions (N = 95, F_(2,93)_ = 2.8, P = 0.09). Flowering density was thus the main determinant of pod density.

**Fig 4:**
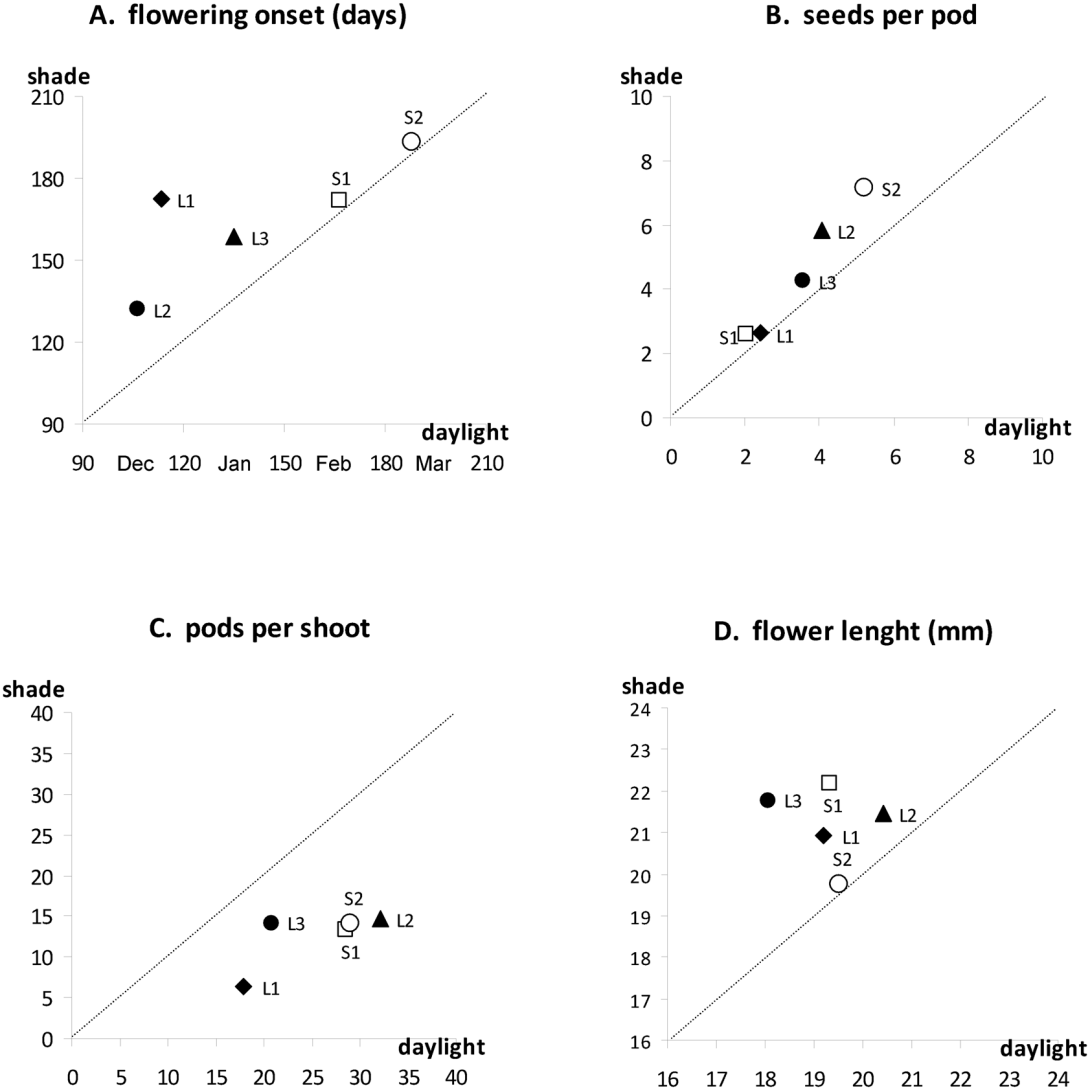
Relation between trait means measured on *Ulex europaeus* in full daylight and in the shade—reproductive traits. Each point represents the average of the values obtained for a given genotype for all years of measurement (4 years for A, B, C, one year for D). Dotted line: X = Y (equal values in daylight and shade).

Shading tended to delay flowering onset, but this effect was more dependent on the year rather than the shading level (Figs 1 and 2). The effect of shading was however predominant for flower length: flowers in the shade were larger than those fully exposed to the sun (Fig 4).

#### Number of seeds per pod and seed predation

**Fig 5:**
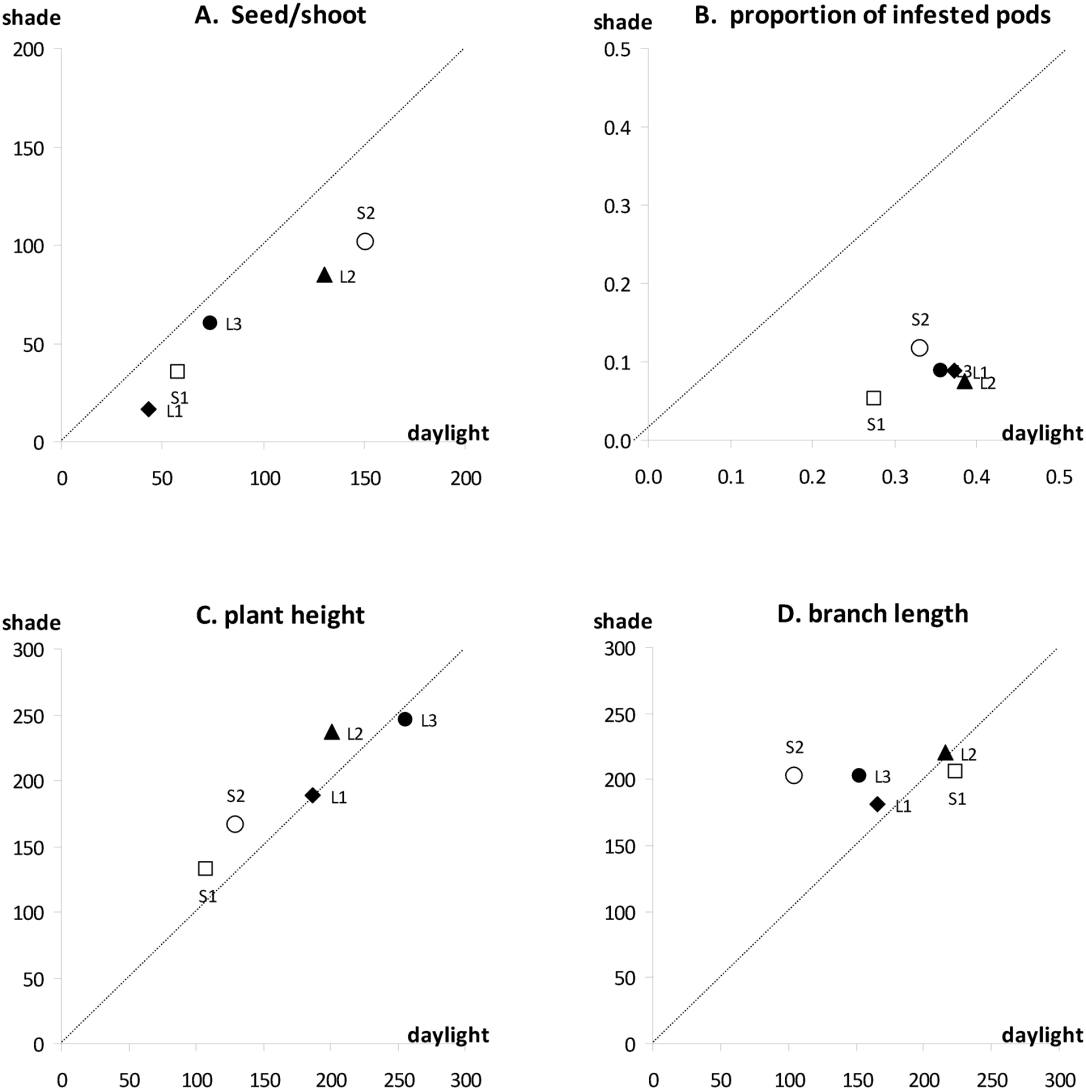
Relation between trait means measured on *Ulex europaeus* in full daylight and in the shade—reproductive and vegetative traits. Each point represents the average of the values obtained for a given genotype for all years of measurement (4 years for A and B, one year for C and D). Dotted line: X = Y (equal values in daylight and shade).

Shading tended to increase the number of seeds per pod: under 65% shade, the increase was considerable in the first year, but was hard to estimate in the second year due to the small number of pods produced (Fig 1). Under 30% shade, the increase in the number of seeds per pod was relatively small in the first year, but reached the values observed under daylight by the end of the experiment (Fig 2). The increase in seeds per pod was however insufficient to compensate for the decrease in pod production, and the overall number of seeds per shoot was lower in the shade than in the light (Fig 5).

Shading tended to decrease the proportion of infested pods: under 65% shade, the percentage of infested pods was very low (0–10%; Fig 1). Under 30% shade, it increased to 5–30%, but remained lower than in daylight (Fig 2).

#### Plant height and width

Plants in the shade were slightly taller and longer than plants in the sun, as demonstrated by the measurements made in 2008/09 (Fig 5). However, the effect of shading on these variables, although significant, was not very large (Table 3).

**Table 3:** ANOVAs made on vegetative and reproductive variables of *Ulex europaeus* plants grown in a common garden under 65% shade and in full daylight, in 2008/09.

2008/09		plant height			plant width			flower length			flower density	
	Df	MS	F	P	MS	F	P	MS	F	P	Chi²	P
genotype	4	254	**45.00**	**<0.001**	166	**28.83**	**<0.001**	257	3.56	<0.05	26.3	<0.001
shade	1	38	6.75	<0.05	22	3.83	0.058	**2792**	**38.58**	**<0.001**	**833.73**	**<0.001**
gen x shade	4	10	1.90	ns	10	1.90	ns	342	4.74	<0.01	22.7	<0.001
residual	38	5.6			5.7			72.4				

N = 48. Df = degree of freedom. MS = Mean Square. Flower density was tested using likelihood ratios for Poisson distributions. The P-value of the total model was P<0.001 for all variables (likelihood ratio test). In bold type, the factor with the greatest influence—ns: not significant and P>0.10.

### Effects of genotype in response to shading

The effect of genotype was significant for almost all of the variables studied, and the interactions between genotype and shade were significant for all variables linked to flower, pod and seed production (Tables 1, 2 and 3). These significant interactions reveal a significant difference in plasticity among genotypes. The level of plasticity, revealed by the ranks of the genotypes in Figs 4 and 5, depended on the trait studied. Genotypes with the same flowering type had similar ranks for two traits: flowering onset (Fig 4A) and infestation rate (Fig 5B): the three long-flowering genotypes responded to shade more than the two short-flowering genotypes.

### Trait response to year-to-year variations

Mean flowering onset varied widely depending on the year, both in the light and in the shade (Figs 1 and 2). The effect of shading was related to the mean date of flowering onset. In the years with the earliest flowering onsets (2007/08 and 2011/12), (i) plants in the light flowered earlier than plants under shade, and (ii) long-flowering genotypes flowered earlier than short-flowering genotypes. In contrast, in the years with the latest flowering onsets (2008/09 and 2009/10) the flowering onset was synchronized for all plants.

To explore the factors that could trigger flowering onset, we tested the relationships between the date of flowering onset and three climatic factors (average temperature, hours of sunshine and precipitation amount per day). We focused on the correlations between climatic factors and the date of flowering onset that explained at least 25% of the variance (R^2^≥0.25). These conditions were met in a single period: between 8^th^ March and 16^th^ May of the year before the reproductive season tested. During this period, there was a correlation between the date of the next flowering onset and temperature (R<-0.5, P<0.001), rainfall (R>0.6, P<0.001) and irradiance (R<-0.6, P<0.001). This meant that the warmer, sunnier and drier the weather during this period, the earlier the flowering onset would be in the next reproductive season.

Year-to-year variation was less important for seed and pod production, for which shading, genotypes and their interactions explained most of the variance (Tables 1 and 2). However, it was important for infestation rate, which exhibited very high variations depending on the year, although plants under shade were always less infested than plants in full daylight (Tables 1 and 2, Figs 1 and 2).

## Discussion

By growing *U*. *europaeus* clones in a common garden, we were able to demonstrate a high level of plasticity in response to shading for most of the reproductive traits studied, and a genetic variability in this level of plasticity. In addition, we observed that the level of plasticity depended on the year, and that the effect of environmental variations was cumulative over time. This suggests that plasticity can influence the lifetime fitness of gorse and be submitted to selection. More generally, our results suggest that flowering induction and the response to environmental factors of perennial species may involve long-term mechanisms acting over several years.

### Response of reproductive traits to shading

The main effect of shading was a reduction of reproductive effort. The decrease of pod production observed under dense shade could have been due to (i) lower flower production, or (ii) lower flower fertilization, resulting from pollen limitation. Here, pod density appeared to be closely correlated to flower density. This implies that reduction in pod production resulted primarily from a decrease in the number of flowers initiated. It is difficult to know whether this decrease results from reduced flower induction or from reduced investment in reproduction [41,42], but the later explanation seems more likely. Indeed, the effect of shading on flower induction appeared to be limited: shading delayed the date of flowering onset, as often observed for both herbaceous and woody species [42, 43], but the differences between plants under shade or in full light were small. Conversely, the effect of shading on plant size and branch length was moderate. Plants under shade were taller than plants in the light, as often seen in response to shade avoidance [44].

The decrease in the number of flowers and pods under shade was accompanied by an increase in flower size and in number of seeds per pods. Both could have resulted from a re-allocation of resources, as observed in many species for seed number [45] or flower size [46]. An increase in flower size may compensate for the decrease in flower production under shade, since flower size is known to influence pollinator attraction and reproductive success [47]. However, under dense shade the reduction in flower production is too strong to be fully compensated by an increase in flower size. Similarly, a decrease in the number of fruits per plant is rarely fully compensated for by an increase in the number of seeds per fruit [48]. In gorse, this compensation is only partial, since at the shoot level, plants under shade produced fewer seeds than plants in the light.

Further compensation can result from the lower level of pod infestation by weevils. Indeed, whatever the level of shading, pods under shade are less infested than pods in the light, a feature that can hardly be explained by the confounding effect of space and shading, since no spatial effect has been detected on gorse grown in full light in the same experimental garden [35]. The reduced pod infestation in the shade can more likely result from the strong phototropism of weevils [22,49,50], or by a reduced attractiveness of plants producing limited quantities of resources [51,52]. Together with the higher number of seeds per pods observed under shade, the lower level of pod infestation may be efficient in reducing the deleterious effect of dense shading on reproductive outcome.

### Temporal variation in trait response

The reduction of pod production observed under dense shade appeared to be both gradual and reversible. Indeed, in the first year under 65% shade, pod production decreased strongly but was maintained, while in the second year, pod production almost ceased. This dramatic effect was however gradually reversible. When shading was reduced to 30%, pod production began to increase in the next reproductive season, and two years later it had reached the same level as in full daylight. This implies that the effect of shading on pod production was only observed above a given threshold, since 30% of shading had no effect on it. The existence of such a threshold is in agreement with the results of [32], who showed that the number of pods per gram of green shoot decreased under dense shade but was maintained in moderate shade.

The delay in flowering onset was small even under dense shade, and did not seem to respond to cumulative effects. It did however depend on the year: the difference between shaded and non-shaded plants was higher in the years when the average flowering onset was early. Conversely, in years when plants flowered later, plants in full daylight initiated their flowers as late as those in the shade. The time of year that seemed to have the strongest effect on the triggering of flowering was March/April of the previous year, when high temperature, abundant sunshine and low rainfall promoted early flowering in the next reproductive season. In *U*. *europaeus*, this critical period corresponds to the peak of flowering and to pod initiation of the previous reproductive season [34], when reproductive effort is at its greatest. The fact that meteorological conditions preceding a given reproductive season can have an effect on flowering triggering has already been shown in annual species [53]. Together with the cumulative effect of years, this suggests that flowering induction in woody perennial species can be the result of a complex combination of parameters over a long period of time.

### Genotypic variation in trait response

For all the reproductive traits studied, the magnitude of the response to dense shade depended on the genotype, showing a genetically based polymorphism of plasticity. A genetic polymorphism of the plasticity of reproductive traits was described in many herbaceous species [54,55], but was considered much more seldom in woody species. Here, the variability observed in the level of plasticity is remarkable considering the small number of genotypes studied, and suggests that the genetic variability for this trait is very high, as observed for most life history traits studied so far in gorse [35,56]. However, no genotype was more plastic than others, since the level of plasticity of a given genotype depended on the trait studied. In particular, plasticity of flowering onset depended on the flowering type: long-flowering genotypes tended to initiate their flowering earlier in the light than in the shade, while short-flowering genotypes showed almost no response to shading. In addition, differences between long and short-flowering genotypes were higher in favorable years, when all plants tended to flower earlier. This feature was observed here on a small number of genotypes, but is in agreement with long-term monitoring of natural populations ([34] and unpublished results), suggesting that the difference between long- and short-flowering genotypes may partly lie in their ability to advance their flowering onset under favorable conditions. Plasticity could thus be an important component of gorse flowering strategies.

### Consequences for *U*. *europaeus* ecology

The response to shading depended on the trait and on the genotype, but was on average remarkably high. Such a high level of plasticity can result from the polyploidy of the species, in link with its invasiveness [57]. A meta-analysis showed that invasive species had greater phenotypic plasticity than native ones [58], but that this plasticity is not always adaptive when resources are limiting. *Ulex europaeus* appeared to cope well with low levels of shading, since no effects on reproductive traits were detected under 30% shade. Moreover, the reduction of pod infestation observed in the shade may induce equivalent or even higher seed production in moderate shade than in full daylight. This adaptive plasticity may explain why this species, although growing preferentially in open environments, can form successful populations in undergrowth [31]. In contrast, the species is not able to cope with dense shade, and under 65% shade, reproduction decreased strongly. Even so, shading had a greater effect on pod density than on plant height, which may indicate an adaptive reallocation of resources between growth and reproduction, as observed in other species [41]. Indeed, in natural populations of *U*. *europaeus*, plants under dense shade do not reproduce but can survive for several years [32], suggesting the maintenance of growth and survival at the expense of reproduction [59,60]. Here we showed that plants that had stopped reproducing under dense shade, can reproduce in following years under sunnier conditions. The ability of *U*. *europaeus* to restrict its reproductive effort when the canopy is too dense, may thus be a "gap-opportunist" strategy [61], allowing it to survive in shaded conditions and be ready to produce a large number of pods if the canopy reopens, as a result of disturbance, for example.

In *U*. *europaeus*, plasticity does not seem to be an alternative to genetic diversity, but a complementary phenomenon in the implementation of adaptation to a changing environment [62]. Together with genetic diversity, plasticity of gorse could be involved in its capacity to grow in contrasting environmental conditions, which may help to explain its invasiveness and its worldwide distribution.

## Supporting Information

S1 Table Dataset used for all figures and statistics. (XLS)

S1 Text Flowering and fruiting stages of *Ulex europaeus*. (DOC)

S2 Text Statistical script of the analyses performed with R. (TXT)
