# Supplementary material for: Phenotypic Plasticity in Reproductive Traits of the Perennial Shrub Ulex europaeus in Response to Shading: A Multi-Year Monitoring of Cultivated Clones
Source: PLoS One. 2015 Sep 18;10(9):e0137500. doi: 10.1371/journal.pone.0137500 (PMC4575064; doi:10.1371/journal.pone.0137500)
Supplement: S1 Text — (DOC) [file pone.0137500.s002.doc]

Flowering and fruiting stages of *Ulex europaeus*

Stage 0 : no buds

Stage 1 : buds < 2 mm long

Stage 2 : most buds 2 to 5 mm long, few buds 5 to 7 mm long

Stage 3 : most buds 5 to 7 mm long, few buds 7 to 10 mm long, few or no flowers

Stage 4 : most buds 7 to 10 mm long, several flowers

Stage 5 : full bloom: many open flowers

Stage 6 : several wilted flowers, with or without pods (small soft pods)

Stage 7 : open and wilted flowers, green hard pods, green seeds with green arils

Stage 8 : very hard pods, green seeds with yellow arils, with or without flowers

Stage 9 : green to brownish pods, yellow seeds, with or without flowers

Stage 10 : green to ripe pods, <1/3 ripe pods

Stage 11 : 1/3 to 2/3 ripe pods, few or no open pods

Stage 12 : >2/3 ripe pods, several open pods

Stage 0 : all pods open
